# Supplementary material for: Novel Insight into the Effects of CpxR on Salmonella enteritidis Cells during the Chlorhexidine Treatment and Non-Stressful Growing Conditions
Source: Int J Mol Sci. 2021 Aug 19;22(16):8938. doi: 10.3390/ijms22168938 (PMC8396259; doi:10.3390/ijms22168938)
Supplement: Supplementary file 1 [file ijms-22-08938-s001.zip › Supplementary/Supplementary Table S1.pdf]

Gene / locus name	Protein Function	Accession Number	Molecular Weight	Permutation Test (p-value) Benjamini-Hochberg (p < 0.02370)	Log2 Fold Change	Fold Change
**dtd**	D-tyrosyl-tRNA(Tyr) deacylase	WP_000560968.1	16 kDa	0.019	3.65	12.55
**PRK00110**	YebC/PmpR family DNA-binding transcriptional regulator	WP_000907242.1	26 kDa	0.004	3.42	10.70
**proA**	Glutamate-5-semialdehyde dehydrogenase	WP_000893239.1	45 kDa	0.004	3.02	8.11
**yeaD**	D-hexose-6-phosphate mutarotase	WP_000608660.1	32 kDa	0.008	3.02	8.11
**rne**	Ribonuclease E	WP_079920831.1	119 kDa	0.004	3	8.00
**yajQ**	YajQ family cyclic di-GMP-binding protein	WP_001539312.1	19 kDa	0.004	2.93	7.62
**HTH_XRE**	Helix-turn-helix domain-containing protein	WP_001705923.1	21 kDa	0.002	2.91	7.52
**rpsG**	30S ribosomal protein S7	WP_001138043.1	18 kDa	0.004	2.74	6.68
	Phage repressor protein	WP_000932273.1	23 kDa	0.013	2.68	6.41
**purL**	Phosphoribosylformylglycinamidine synthase	WP_000970028.1	141 kDa	0.002	2.67	6.36
**mod**	Site-specific DNA-methyltransferase	WP_000910353.1	73 kDa	0.003	2.39	5.24
**cpxA**	Envelope stress sensor histidine kinase CpxA	WP_000580398.1	52 kDa	0.002	2.37	5.17
	Type I restriction-modification protein specificity subunit	WP_000863534.1	48 kDa	0.004	2.34	5.06
**clpB**	ATP-dependent chaperone ClpB	WP_024156384.1	95 kDa	0.004	2.32	4.99
**fliB**	Flagellin lysine-N-methylase	WP_001707899.1	45 kDa	0.004	2.32	4.99
**mukB**	Chromosome partition protein MukB	WP_000572746.1	170 kDa	0.014	2.32	4.99
**COG4453**	DUF1778 domain-containing protein	WP_001110450.1	11 kDa	0.002	2.31	4.96
**DUF1496**	DUF1496 domain-containing protein	WP_000336848.1	10 kDa	0.004	2.28	4.86
**DUF2732**	DUF2732 family protein	WP_001244219.1	9 kDa	0.004	2.24	4.72
**PRK10605**	Alkene reductase	WP_000092935.1	40 kDa	0.004	2.19	4.56
**fliC**	Flagellin FliC	WP_094204704.1	53 kDa	0.004	2.18	4.53
**relB**	Type II toxin-antitoxin system RelB/DinJ family antitoxin	WP_000729713.1	9 kDa	0.004	2.15	4.44
**DUF1481**	DUF1481 domain-containing protein	WP_001083931.1	26 kDa	0.004	2.14	4.41
**DUF2511**	DUF2511 domain-containing protein	WP_000722370.1	13 kDa	0.00024	2.07	4.20
**vapB**	Toxin-antitoxin system antitoxin VapB	WP_031606523.1	9 kDa	0.002	1.94	3.84
**traM**	Relaxosome protein TraM	WP_079983192.1	14 kDa	0.004	1.93	3.81
	Hypothetical protein	WP_000556389.1	35 kDa	0.001	1.81	3.51
**ptsP**	Phosphoenolpyruvate--protein phosphotransferase	WP_079829802.1	85 kDa	0.003	1.79	3.46
**nfo**	Deoxyribonuclease IV	WP_001729752.1	31 kDa	0.004	1.42	2.68
**dsbA**	Thioredoxin domain-containing protein	WP_079957070.1	24 kDa	0.004	1.35	2.55
**prmC**	Peptide chain release factor N(5)-glutamine methyltransferase	WP_000347310.1	31 kDa	0.002	1.25	2.38
**PRK01076**	L-rhamnose isomerase	WP_000211477.1	48 kDa	0.004	1.23	2.35
**SR_ResInv**	Recombinase family protein	WP_001240331.1	21 kDa	0.0058	1.21	2.31
**PRK11118**	Monooxygenase	WP_000047689.1	12 kDa	0.006	1.16	2.23
**ycfS**	L,D-transpeptidase family protein	WP_001708681.1	33 kDa	0.002	1.15	2.22
**dinG**	ATP-dependent DNA helicase DinG	WP_001218636.1	81 kDa	0.008	1.14	2.20
**plaP**	Putrescine/proton symporter PlaP	WP_000178807.1	50 kDa	0.003	1.13	2.19
**ybbK**	DUF1722 domain-containing protein	WP_138017663.1	36 kDa	0.004	1.13	2.19
**moaA**	GTP 3',8-cyclase MoaA	WP_000168181.1	37 kDa	0.004	1.03	2.04
**narI**	Respiratory nitrate reductase subunit gamma	WP_001777945.1	11 kDa	0.008	1	2.00

Gene / locus name	Protein Function	Accession Number	Molecular Weight	Permutation Test (p-value) Benjamini-Hochberg (p < 0.02370)	Log2 Fold Change	Fold Change
**tdcB**	Bifunctional threonine ammonia-lyase/L-serine ammonia-lyase TdcB	WP_148523547.1	35 kDa	0.002	0.86	1.82
**melR**	Transcriptional regulator MelR	WP_000106992.1	36 kDa	0.003	0.83	1.78
**ppiA**	Peptidylprolyl isomerase A	WP_076916862.1	20 kDa	0.004	0.83	1.78
**yajR**	MFS transporter	WP_023227330.1	49 kDa	0.002	0.82	1.77
**ppnP**	Pyrimidine/purine nucleoside phosphorylase	WP_000941950.1	10 kDa	0.004	0.82	1.77
**gntT**	Gluconate transporter	WP_001131737.1	46 kDa	0.012	0.82	1.77
**qseG**	Two-component system QseEF-associated lipoprotein QseG	WP_001054239.1	28 kDa	0.004	0.81	1.75
**hypC**	Hydrogenase 3 maturation protein HypC	WP_022742810.1	10 kDa	0.004	0.81	1.75
**hpf**	Ribosome hibernation promoting factor	WP_061104299.1	11 kDa	0.016	0.81	1.75
**PRK11653**	DUF1190 family protein	WP_000831520.1	23 kDa	0.001	0.8	1.74
**sufB**	Fe-S cluster assembly protein SufB	WP_000089374.1	55 kDa	0.00055	0.79	1.73
**pflD**	Formate C-acetyltransferase	WP_000149710.1	86 kDa	0.002	0.79	1.73
**DUF4432**	DUF4432 family protein	WP_000213211.1	38 kDa	0.002	0.79	1.73
**malS**	Alpha-amylase	WP_000761323.1	76 kDa	0.004	0.78	1.72
**ccdB**	Type II toxin-antitoxin system toxin CcdB	WP_001159863.1	12 kDa	0.004	0.77	1.71
**nirC**	Nitrite transporter NirC	WP_000493575.1	29 kDa	0.004	0.76	1.69
**PRK10556**	DUF2002 family protein	WP_000281305.1	13 kDa	0.001	0.75	1.68
**DCR_FMN**	NADPH-dependent 2,4-dienoyl-CoA reductase	WP_001728593.1	73 kDa	0.0034	0.74	1.67
**menA**	o-succinylbenzoate--CoA ligase	WP_000144664.1	50 kDa	0.00086	0.73	1.66
**algH**	YqgE/AlgH family protein	WP_001053173.1	21 kDa	0.001	0.73	1.66
**mglA**	Galactose/methyl galactoside ABC transporter ATP-binding protein MglA	WP_000535907.1	56 kDa	0.004	0.71	1.64
**paaY**	Gamma carbonic anhydrase family protein	WP_001285631.1	20 kDa	0.002	0.7	1.62
**rocR**	Sigma-54-dependent transcriptional regulator	WP_000168923.1	53 kDa	0.004	0.7	1.62
**narJ**	Nitrate reductase molybdenum cofactor assembly chaperone	WP_000571667.1	26 kDa	0.02	0.7	1.62
**ilvB**	Acetolactate synthase large subunit	WP_001734390.1	55 kDa	0.002	0.68	1.60
**cyaY**	Iron donor protein CyaY	WP_000999925.1	12 kDa	0.003	0.68	1.60
**raiA**	Ribosome-associated translation inhibitor RaiA	WP_000178449.1	13 kDa	0.00015	0.67	1.59
**pdxA**	D-threonate 4-phosphate dehydrogenase	WP_000448745.1	35 kDa	0.002	0.67	1.59
**aceF**	Pyruvate dehydrogenase complex dihydrolipoyllysine-residue acetyltransferase	WP_000963608.1	66 kDa	0.004	0.67	1.59
**dkgB**	2,5-didehydrogluconate reductase DkgB	WP_000154878.1	29 kDa	0.009	0.67	1.59
**mliC**	C-type lysozyme inhibitor	WP_001132086.1	13 kDa	0.002	0.66	1.58
**rfaC**	Lipopolysaccharide heptosyltransferase RfaC	WP_076915342.1	35 kDa	0.00031	0.65	1.57
**argR**	Arginine repressor	WP_000666051.1	18 kDa	0.002	0.65	1.57
**PRK00124**	YaiI/YqxD family protein	WP_001708868.1	19 kDa	0.0029	0.65	1.57
**yajL**	Protein deglycase YajL	WP_001275803.1	21 kDa	0.001	0.63	1.55
**lysA**	Diaminopimelate decarboxylase	WP_080208216.1	38 kDa	0.0012	0.63	1.55
**queC**	7-cyano-7-deazaguanine synthase QueC	WP_135426189.1	22 kDa	0.004	0.63	1.55
**dnaC**	DNA replication protein DnaC	WP_000799921.1	28 kDa	0.001	0.62	1.54
**mviM**	Gfo/Idh/MocA family oxidoreductase	WP_001259728.1	34 kDa	0.004	0.61	1.53
**ulaA**	PTS ascorbate transporter subunit IIC	WP_001721663.1	47 kDa	0.004	0.58	1.49

Gene / locus name	Protein Function	Accession Number	Molecular Weight	Permutation Test (p-value) Benjamini-Hochberg (p < 0.02370)	Log2 Fold Change	Fold Change
**hisP**	Histidine ABC transporter ATP-binding protein HisP	WP_000986780.1	29 kDa	0.012	0.58	1.49
**hlyD**	secretion protein HlyD	WP_080199248.1	36 kDa	0.00013	0.57	1.48
**tas**	Aldo/keto reductase	WP_000782140.1	36 kDa	0.007	0.57	1.48
**PRK05090**	YggU family protein	WP_080202365.1	8 kDa	0.008	0.57	1.48
**cysI**	Assimilatory sulfite reductase (NADPH) hemoprotein subunit	WP_079983308.1	64 kDa	0.018	0.57	1.48
**argC**	N-acetyl-gamma-glutamyl-phosphate reductase	WP_000935334.1	36 kDa	0.00017	0.56	1.47
**yjjG**	Pyrimidine 5'-nucleotidase	WP_000978807.1	25 kDa	0.004	0.55	1.46
**uxaC**	Glucuronate isomerase	WP_000190176.1	53 kDa	0.00021	0.54	1.45
**ydfG**	Bifunctional NADP-dependent 3-hydroxy acid dehydrogenase/3-hydroxypropionate dehydrogenase YdfG	WP_000636564.1	27 kDa	0.0038	0.54	1.45
**lpxO**	Lipid A hydroxylase LpxO	WP_000457031.1	35 kDa	0.012	0.54	1.45
**mgrA**	L-glyceraldehyde 3-phosphate reductase	WP_020899059.1	37 kDa	0.015	0.54	1.45
**melA**	Alpha-galactosidase	WP_001520868.1	51 kDa	0.005	0.53	1.44
**pspF**	Phage shock protein operon transcriptional activator	WP_000807306.1	37 kDa	0.007	0.53	1.44
**argE**	Acetylornithine deacetylase	WP_000800208.1	42 kDa	0.008	0.53	1.44
**dcp**	peptidyl-dipeptidase Dcp	WP_000685856.1	76 kDa	0.00019	0.52	1.43
**garR**	2-hydroxy-3-oxopropionate reductase	WP_000178103.1	31 kDa	0.002	0.52	1.43
**potF**	Spermidine/putrescine ABC transporter substrate-binding protein PotF	WP_000125769.1	41 kDa	0.004	0.52	1.43
**glk**	Glucokinase	WP_000170371.1	35 kDa	0.00015	0.51	1.42
**rnd**	Ribonuclease D	WP_001109121.1	42 kDa	0.001	0.51	1.42
**fxsA**	Membrane protein FxsA	WP_001541341.1	19 kDa	0.002	0.51	1.42
**DUF2500**	DUF2500 domain-containing protein	WP_000042859.1	14 kDa	0.004	0.51	1.42
**PRK10941**	Tetratricopeptide repeat-containing protein	WP_001257065.1	31 kDa	0.00015	0.5	1.41
**PRK10718**	RpoE-regulated lipoprotein	WP_000838971.1	21 kDa	0.001	0.5	1.41
**cysE**	Serine O-acetyltransferase	WP_135425361.1	25 kDa	0.0079	0.5	1.41
	Hypothetical protein	WP_000587738.1	27 kDa	0.001	0.49	1.40
**rihA**	Pyrimidine-specific ribonucleoside hydrolase RihA	WP_001207422.1	34 kDa	0.002	0.48	1.39
**cydC**	Cysteine/glutathione ABC transporter ATP-binding protein/permease CydC	WP_001202251.1	63 kDa	0.0055	0.48	1.39
**nrdE**	Class 1b ribonucleoside-diphosphate reductase subunit alpha	WP_000246182.1	81 kDa	0.01	0.48	1.39
**creB**	Two-component system response regulator CreB	WP_001187038.1	26 kDa	0.004	0.47	1.39
**yejA**	ABC transporter substrate-binding protein	WP_135416368.1	69 kDa	0.0049	0.47	1.39
**htpX**	Protease HtpX	WP_001722428.1	32 kDa	0.013	0.47	1.39
**hypB**	Hydrogenase nickel incorporation protein HypB	WP_135423778.1	25 kDa	0.00015	0.46	1.38
**iclR**	IclR family transcriptional regulator	WP_135415874.1	26 kDa	0.003	0.46	1.38
**bcsQ**	Cellulose biosynthesis protein BcsQ	WP_000996122.1	27 kDa	0.004	0.45	1.37
**PRK09248**	Phosphatase	WP_000283638.1	27 kDa	0.001	0.44	1.36
**fdxH**	Formate dehydrogenase subunit beta	WP_051129234.1	33 kDa	0.001	0.44	1.36
**ycaL**	M48 family metallopeptidase	WP_000701824.1	27 kDa	0.001	0.44	1.36
**zinT**	Metal-binding protein ZinT	WP_000234685.1	25 kDa	0.001	0.44	1.36
**iscR**	Fe-S cluster assembly transcriptional regulator IscR	WP_001241346.1	17 kDa	0.0037	0.44	1.36
**hiT**	HIT family protein	WP_000128238.1	17 kDa	0.004	0.44	1.36

Gene / locus name	Protein Function	Accession Number	Molecular Weight	Permutation Test (p-value) Benjamini-Hochberg (p < 0.02370)	Log2 Fold Change	Fold Change
**rhmD**	L-rhamnonate dehydratase	WP_139330018.1	44 kDa	0.006	0.44	1.36
**yhhY**	N-acetyltransferase	WP_001290280.1	19 kDa	0.014	0.44	1.36
**grxA**	GrxA family glutaredoxin	WP_076916967.1	10 kDa	0.001	0.43	1.35
**hexR**	Transcriptional regulator HexR	WP_140011305.1	32 kDa	0.0014	0.43	1.35
**lolD**	Lipoprotein-releasing ABC transporter ATP-binding protein LolD	WP_001033714.1	25 kDa	0.004	0.43	1.35
**dmsB**	Dimethylsulfoxide reductase subunit B	WP_132631290.1	55 kDa	0.00045	0.42	1.34
**menC**	o-succinylbenzoate synthase	WP_001255561.1	35 kDa	0.003	0.42	1.34
**ubiJ**	Ubiquinone biosynthesis protein UbiJ	WP_001116090.1	22 kDa	0.009	0.42	1.34
**tolQ**	Tol-Pal system protein TolQ	WP_000131318.1	26 kDa	0.001	0.41	1.33
**PRK08317**	Class I SAM-dependent methyltransferase	WP_135416073.1	19 kDa	0.002	0.41	1.33
**yqjE**	Membrane protein	WP_000785626.1	15 kDa	0.002	0.41	1.33
**rimJ**	30S ribosomal protein S5 alanine N-acetyltransferase	WP_000468201.1	23 kDa	0.004	0.41	1.33
**hisC**	Histidinol-phosphate transaminase	WP_000102712.1	40 kDa	0.007	0.41	1.33
**hemD**	Uroporphyrinogen-III synthase	WP_000025491.1	28 kDa	0.018	0.41	1.33
**clpA**	ATP-dependent chaperone Clp protease ATP-binding subunite ClpA	WP_000934064.1	84 kDa	0.00027	0.4	1.32
**lpxA**	Acyl-ACP--UDP-N-acetylglucosamine O-acyltransferase	WP_000565950.1	28 kDa	0.003	0.4	1.32
**emrA**	HlyD family secretion protein	WP_077948222.1	33 kDa	0.003	0.4	1.32
**hiuH**	Hydroxyisourate hydrolase	WP_080199438.1	15 kDa	0.004	0.4	1.32
**mltC**	Membrane-bound lytic murein transglycosylase MltC	WP_000976287.1	40 kDa	0.006	0.4	1.32
**rffM**	Lipopolysaccharide N-acetylmannosaminouronosyltransferase	WP_000183613.1	28 kDa	0.012	0.4	1.32
**yjjP**	Threonine/serine exporter ThrE family protein	WP_001674846.1	28 kDa	0.016	0.4	1.32
**mmsB**	NAD(P)-dependent oxidoreductase	WP_000206956.1	31 kDa	0.002	0.39	1.31
**dipZ**	Protein-disulfide reductase DsbD	WP_141124765.1	64 kDa	0.002	0.39	1.31
**PRK05421**	Endonuclease/exonuclease/phosphatase family protein	WP_001230964.1	30 kDa	0.004	0.39	1.31
**fucU**	L-fucose mutarotase	WP_000920845.1	15 kDa	0.009	0.39	1.31
**rssB**	Two-component system response regulator RssB	WP_000193429.1	37 kDa	0.012	0.39	1.31
**dcyD**	D-cysteine desulfhydrase	WP_001128180.1	35 kDa	0.002	0.38	1.30
**gltI**	Amino acid ABC transporter substrate-binding protein	WP_000588819.1	34 kDa	0.003	0.38	1.30
**rsmE**	16S rRNA (uracil(1498)-N(3))-methyltransferase	WP_135424967.1	20 kDa	0.004	0.38	1.30
**rffC**	dTDP-4-amino-4,6-dideoxy-D-galactose acyltransferase	WP_001145156.1	25 kDa	0.004	0.38	1.30
**aroE**	Shikimate dehydrogenase	WP_000451193.1	29 kDa	0.00011	0.37	1.29
**metN**	Methionine ABC transporter ATP-binding protein MetN	WP_079837681.1	32 kDa	0.00018	0.37	1.29
**cysQ**	3'(2'),5'-bisphosphate nucleotidase CysQ	WP_000893398.1	27 kDa	0.00028	0.37	1.29
**pepT**	Peptidase T	WP_000359414.1	45 kDa	0.001	0.37	1.29
**Fdh-alpha**	Formate dehydrogenase N subunit alpha	WP_010989279.1	79 kDa	0.001	0.37	1.29
**yhdP**	AsmA2 domain-containing protein	WP_001713664.1	138 kDa	0.0014	0.37	1.29
**ytfJ**	YtfJ family protein	WP_000175301.1	21 kDa	0.002	0.37	1.29
**yfcL**	YfcL family protein	WP_000559749.1	10 kDa	0.002	0.37	1.29
**lptF**	LPS export ABC transporter permease LptF	WP_000584130.1	40 kDa	0.005	0.37	1.29
**ribB**	3,4-dihydroxy-2-butanone-4-phosphate synthase	WP_001076978.1	23 kDa	0.01	0.37	1.29

Gene / locus name	Protein Function	Accession Number	Molecular Weight	Permutation Test (p-value) Benjamini-Hochberg (p < 0.02370)	Log2 Fold Change	Fold Change
**yfhB**	Phosphatidylglycerophosphatase C	WP_000253558.1	24 kDa	0.017	0.37	1.29
**serA**	Phosphoglycerate dehydrogenase	WP_001151621.1	44 kDa	0.018	0.37	1.29
**asmA**	Outer membrane assembly protein AsmA	WP_001252257.1	69 kDa	0.00037	0.36	1.28
**pdxJ**	Pyridoxine 5'-phosphate synthase	WP_000818964.1	26 kDa	0.001	0.36	1.28
**tldD**	Metalloprotease TldD	WP_000055936.1	51 kDa	0.001	0.36	1.28
**ilvE**	Branched-chain-amino-acid transaminase	WP_000208528.1	34 kDa	0.001	0.36	1.28
**glrR**	Two-component system response regulator GlrR	WP_000625590.1	49 kDa	0.002	0.36	1.28
**djlA**	Co-chaperone DjlA	WP_001200595.1	31 kDa	0.002	0.36	1.28
**PRK01722**	Formimidoylglutamase	WP_000195682.1	34 kDa	0.002	0.36	1.28
**exbB**	Biopolymer transporter ExbB	WP_000527859.1	26 kDa	0.004	0.36	1.28
**aroL**	Shikimate kinase AroL	WP_000983565.1	20 kDa	0.004	0.36	1.28
**rseC**	SoxR-reducing system protein RseC	WP_000589049.1	17 kDa	0.018	0.36	1.28
**adeB**	Multidrug efflux RND transporter permease subunit	WP_001132506.1	114 kDa	0.001	0.35	1.27
**sapA**	Peptide ABC transporter substrate-binding protein SapA	WP_001241619.1	62 kDa	0.001	0.35	1.27
**aegA**	Oxidoreductase FeS-binding subunit	WP_001078922.1	72 kDa	0.008	0.35	1.27
**murB**	UDP-N-acetylmuramate dehydrogenase	WP_000149793.1	38 kDa	0.00012	0.34	1.27
**cobK**	Cobalt-precorrin-6A reductase	WP_023226669.1	28 kDa	0.00032	0.34	1.27
**mglB**	Galactose/glucose ABC transporter substrate-binding protein MglB	WP_069057568.1	36 kDa	0.00056	0.34	1.27
**hisG**	ATP phosphoribosyltransferase	WP_000886600.1	33 kDa	0.0007	0.34	1.27
**fumB**	Fumarate hydratase	WP_000066590.1	60 kDa	0.001	0.34	1.27
**modF**	Molybdate ABC transporter ATP-binding protein ModF	WP_079983686.1	55 kDa	0.001	0.34	1.27
**rraA**	Ribonuclease E activity regulator RraA	WP_000872918.1	17 kDa	0.002	0.34	1.27
**secF**	Protein translocase subunit SecF	WP_000046629.1	35 kDa	0.0037	0.34	1.27
**leuA**	2-isopropylmalate synthase	WP_000082813.1	57 kDa	0.004	0.34	1.27
**rseA**	Anti-sigma-E factor RseA	WP_001168374.1	24 kDa	0.023	0.34	1.27
**ygfZ**	tRNA-modifying protein YgfZ	WP_000874169.1	36 kDa	0.00011	0.33	1.26
**tamA**	Autotransporter assembly complex protein TamA	WP_001120233.1	65 kDa	0.00016	0.33	1.26
**radA**	DNA repair protein RadA	WP_001029705.1	49 kDa	0.001	0.33	1.26
**ddl**	D-alanine--D-alanine ligase B	WP_000763905.1	33 kDa	0.001	0.33	1.26
**ftsE**	Cell division ATP-binding protein FtsE	WP_000617729.1	24 kDa	0.002	0.33	1.26
**hinT**	Purine nucleoside phosphoramidase	WP_001532068.1	13 kDa	0.004	0.33	1.26
**DLH**	Dienelactone hydrolase family protein	WP_000213955.1	29 kDa	0.004	0.33	1.26
**greA**	Transcription elongation factor GreA	WP_040133551.1	18 kDa	0.0044	0.33	1.26
**lptG**	LPS export ABC transporter permease LptG	WP_001182241.1	40 kDa	0.009	0.33	1.26
**rffG**	dTDP-glucose 4,6-dehydratase	WP_000822139.1	40 kDa	0.015	0.33	1.26
**rfbC**	dTDP-4-dehydrorhamnose 3,5-epimerase	WP_079928519.1	21 kDa	0.021	0.33	1.26
**queF**	NADPH-dependent 7-cyano-7-deazaguanine reductase QueF	WP_000100459.1	33 kDa	0.00023	0.32	1.25
**gudB**	Glucarate dehydratase	WP_000107113.1	49 kDa	0.00036	0.32	1.25
**luxS**	S-ribosylhomocysteine lyase	WP_001130193.1	19 kDa	0.00067	0.32	1.25
**lpoB**	Penicillin-binding protein activator LpoB	WP_000164353.1	23 kDa	0.002	0.32	1.25

Gene / locus name	Protein Function	Accession Number	Molecular Weight	Permutation Test (p-value) Benjamini-Hochberg (p < 0.02370)	Log2 Fold Change	Fold Change
**HTH_1**	LysR family transcriptional regulator	WP_001746645.1	34 kDa	0.004	0.32	1.25
**argP**	LysR family transcriptional regulator ArgP	WP_000828344.1	33 kDa	0.004	0.32	1.25
**rlmE**	23S rRNA (uridine(2552)-2'-O)-methyltransferase RlmE	WP_079785907.1	23 kDa	0.005	0.32	1.25
**eptA**	Phosphoethanolamine transferase EptA	WP_038427769.1	62 kDa	0.013	0.32	1.25
**sufS**	Cysteine desulfurase SufS	WP_000143845.1	45 kDa	0.013	0.32	1.25
**selB**	Selenocysteine-specific translation elongation factor	WP_000582387.1	69 kDa	0.0004	0.31	1.24
**ybfF**	Esterase	WP_079971262.1	29 kDa	0.00069	0.31	1.24
**miaB**	tRNA (N6-isopentenyl adenosine(C2)-methylthiotransferase MiaB	WP_001519200.1	54 kDa	0.00077	0.31	1.24
**draG**	ADP-ribosylglycohydrolase family protein	WP_000557872.1	37 kDa	0.001	0.31	1.24
**trxC**	Thioredoxin TrxC	WP_001098732.1	16 kDa	0.002	0.31	1.24
**ldcA**	Muramoyltetrapeptide carboxypeptidase	WP_000051603.1	33 kDa	0.00078	0.3	1.23
**fucI**	L-fucose isomerase	WP_108630795.1	65 kDa	0.001	0.3	1.23
**yjfP**	Esterase	WP_000560307.1	27 kDa	0.001	0.3	1.23
**chbA**	PTS N,N'-diacetylchitobiose transporter subunit IIA	WP_001732541.1	11 kDa	0.001	0.3	1.23
**nadE**	Ammonia-dependent NAD(+) synthetase	WP_000174977.1	30 kDa	0.002	0.3	1.23
**degS**	Outer membrane-stress sensor serine endopeptidase DegS	WP_000497705.1	38 kDa	0.002	0.3	1.23
**PRK11768**	Serine/threonine protein kinase	WP_000999265.1	38 kDa	0.003	0.3	1.23
**dapB**	4-hydroxy-tetrahydrodipicolinate reductase	WP_076915666.1	29 kDa	0.005	0.3	1.23
**lpxC**	UDP-3-O-acyl-N-acetylglucosamine deacetylase	WP_000595474.1	34 kDa	0.012	0.3	1.23
**nrfF**	Aspartate-semialdehyde dehydrogenase	WP_076916980.1	36 kDa	0.014	0.3	1.23
**proV**	Glycine betaine/L-proline ABC transporter ATP-binding protein ProV	WP_069057539.1	44 kDa	0.017	0.3	1.23
**slyX**	Protein SlyX	WP_001152702.1	8 kDa	0.017	0.3	1.23
**nudC**	NAD(+) diphosphatase	WP_000373954.1	30 kDa	0.021	0.3	1.23
**holA**	DNA polymerase III subunit delta	WP_000620494.1	39 kDa	0.022	0.3	1.23
**znuC**	Zinc ABC transporter ATP-binding protein ZnuC	WP_000203014.1	28 kDa	0.001	0.29	1.22
**macB**	Macrolide ABC transporter ATP-binding protein/permease MacB	WP_000125899.1	71 kDa	0.003	0.29	1.22
**ycjF**	TIGR01620 family protein	WP_079786002.1	39 kDa	0.0041	0.29	1.22
**pfkA**	ATP-dependent 6-phosphofructokinase	WP_000591793.1	35 kDa	0.005	0.29	1.22
**dnaQ**	DNA polymerase III subunit epsilon	WP_031616632.1	28 kDa	0.013	0.29	1.22
**tehB**	Tellurite resistance methyltransferase TehB	WP_079920847.1	22 kDa	0.023	0.29	1.22
**msrB**	Peptide-methionine (R)-S-oxide reductase MsrB	WP_000102941.1	17 kDa	0.023	0.29	1.22
**glpR**	DeoR/GlpR transcriptional regulator	WP_000059693.1	29 kDa	0.00037	0.28	1.21
**qseB**	Two-component system response regulator QseB	WP_001221574.1	24 kDa	0.002	0.28	1.21
**cueO**	Multicopper oxidase CueO	WP_000946053.1	59 kDa	0.003	0.28	1.21
**fetA**	Iron ABC transporter ATP-binding protein FetA	WP_000166987.1	25 kDa	0.004	0.28	1.21
**ssb**	Single-stranded DNA-binding protein SSB1	WP_000168322.1	19 kDa	0.01	0.28	1.21
**lpxL**	LpxL/LpxP family Kdo(2)-lipid IV(A) lauroyl/palmitoleoyl acyltransferasee	WP_000163977.1	35 kDa	0.01	0.28	1.21
**lexA**	Repressor LexA	WP_000646075.1	22 kDa	0.014	0.28	1.21
**truA**	tRNA pseudouridine(38-40) synthase TruA	WP_000016626.1	30 kDa	0.019	0.28	1.21
**rhlB**	ATP-dependent RNA helicase RhlB	WP_000047524.1	47 kDa	0.001	0.27	1.21

Gene / locus name	Protein Function	Accession Number	Molecular Weight	Permutation Test (p-value) Benjamini-Hochberg (p < 0.02370)	Log2 Fold Change	Fold Change
**selA**	L-seryl-tRNA(Sec) selenium transferase	WP_000200193.1	51 kDa	0.001	0.27	1.21
**murF**	UDP-N-acetylmuramoyl-tripeptide--D-alanyl-D-alanine ligase	WP_000626630.1	48 kDa	0.002	0.27	1.21
**miaA**	tRNA (adenosine(37)-N6)-dimethylallyltransferase MiaA	WP_066038542.1	35 kDa	0.002	0.27	1.21
**pncC**	Nicotinamide-nucleotide amidase	WP_001728877.1	18 kDa	0.008	0.27	1.21
**ridA**	2-iminobutanoate/2-iminopropanoate deaminase	WP_000047544.1	14 kDa	0.00018	0.26	1.20
**sufC**	Fe-S cluster assembly ATPase SufC	WP_001580259.1	28 kDa	0.0004	0.26	1.20
**mnmH**	tRNA 2-selenouridine synthase MnmH	WP_001154079.1	41 kDa	0.001	0.26	1.20
**cyaA**	Class I adenylate cyclase	WP_001708418.1	97 kDa	0.002	0.26	1.20
**csdE**	Cysteine desulfurase sulfur acceptor subunit CsdE	WP_000184198.1	16 kDa	0.003	0.26	1.20
**ampD**	1,6-anhydro-N-acetylmuramyl-L-alanine amidase AmpD	WP_000936324.1	21 kDa	0.003	0.26	1.20
**PRK11587**	Sugar phosphatase	WP_077942669.1	24 kDa	0.006	0.26	1.20
**frsA**	Esterase FrsA	WP_000189582.1	47 kDa	0.008	0.26	1.20
**entF**	Enterobactin non-ribosomal peptide synthetase EntF	WP_000194133.1	142 kDa	0.01	0.26	1.20
**nfuA**	Fe-S biogenesis protein NfuA	WP_000619387.1	21 kDa	0.018	0.26	1.20
**nudH**	RNA pyrophosphohydrolase	WP_004245524.1	21 kDa	0.019	0.26	1.20
**bcsB**	Cellulose biosynthesis cyclic di-GMP-binding regulatory protein BcsB	WP_000823808.1	84 kDa	0.002	0.25	1.19
**hipB**	Helix-turn-helix domain-containing protein	WP_049795274.1	20 kDa	0.0034	0.25	1.19
**holC**	DNA polymerase III subunit chi	WP_077952198.1	18 kDa	0.005	0.25	1.19
**tonB**	TonB system transport protein TonB	WP_001517937.1	26 kDa	0.006	0.25	1.19
**apaH**	Bis(5'-nucleosyl)-tetraphosphatase (symmetrical)	WP_000257211.1	31 kDa	0.007	0.25	1.19
**dgt**	dGTPase	WP_000146443.1	60 kDa	0.01	0.25	1.19
**menB**	1,4-dihydroxy-2-naphthoyl-CoA synthase	WP_000640013.1	32 kDa	0.0002	0.24	1.18
**ilvD**	Dihydroxy-acid dehydratase	WP_001127431.1	66 kDa	0.001	0.24	1.18
**degP**	Serine endoprotease DegP	WP_000753958.1	49 kDa	0.001	0.24	1.18
**tcdA**	tRNA cyclic N6-threonylcarbamoyladenosine synthase TcdA	WP_000117742.1	29 kDa	0.001	0.24	1.18
**rcsB**	Transcriptional regulator RcsB	WP_063887130.1	24 kDa	0.003	0.24	1.18
**aroF**	3-deoxy-7-phosphoheptulonate synthase AroF	WP_001168062.1	39 kDa	0.0039	0.24	1.18
**fabZ**	3-hydroxyacyl-ACP dehydratase FabZ	WP_000210741.1	17 kDa	0.004	0.24	1.18
**mukE**	Chromosome partition protein MukE	WP_000060024.1	27 kDa	0.004	0.24	1.18
**lpoA**	Penicillin-binding protein activator	WP_135416286.1	73 kDa	0.011	0.24	1.18
**lpp**	Major outer membrane lipoprotein	WP_001082307.1	8 kDa	0.021	0.24	1.18
**rplU**	50S ribosomal protein L21	WP_000271396.1	12 kDa	0.023	0.24	1.18
**xthA**	Exodeoxyribonuclease III	WP_000673950.1	31 kDa	0.00091	0.23	1.17
**narL**	Two-component system response regulator NarL	WP_038424379.1	24 kDa	0.003	0.23	1.17
**topB**	DNA topoisomerase III	WP_001235866.1	73 kDa	0.0048	0.23	1.17
**bisC**	Molybdopterin guanine dinucleotide-containing S/N-oxide reductase	WP_023227718.1	86 kDa	0.006	0.23	1.17
**mfd**	Transcription-repair coupling factor	WP_001114323.1	130 kDa	0.007	0.23	1.17
**epmA**	Elongation factor P--(R)-beta-lysine ligase	WP_000004789.1	37 kDa	0.008	0.23	1.17
**ispA**	(2E,6E)-farnesyl diphosphate synthase	WP_135415798.1	32 kDa	0.00041	0.22	1.16
**gshB**	Glutathione synthase	WP_000593248.1	35 kDa	0.00092	0.22	1.16

Gene / locus name	Protein Function	Accession Number	Molecular Weight	Permutation Test (p-value) Benjamini-Hochberg (p < 0.02370)	Log2 Fold Change	Fold Change
**ahpC**	Alkyl hydroperoxide reductase subunit C	WP_000052802.1	21 kDa	0.001	0.22	1.16
**ubiK**	Accessory factor UbiK family protein	WP_000566824.1	11 kDa	0.002	0.22	1.16
**arsC**	Arsenate reductase (glutaredoxin)	WP_038437172.1	13 kDa	0.0037	0.22	1.16
**DUF3053**	DUF3053 domain-containing protein	WP_000190523.1	22 kDa	0.005	0.22	1.16
**gorA**	Glutathione-disulfide reductase	WP_000160771.1	49 kDa	0.01	0.22	1.16
**fmt**	Methionyl-tRNA formyltransferase	WP_001285171.1	34 kDa	0.017	0.22	1.16
**avtA**	Valine--pyruvate transaminase	WP_000144381.1	47 kDa	0.022	0.22	1.16
**rfbN**	O antigen biosynthesis rhamnosyltransferase RfbN	WP_000705151.1	36 kDa	0.00012	0.21	1.16
**trnD**	tRNA (guanosine(37)-N1)-methyltransferase TrmD	WP_085419208.1	28 kDa	0.00051	0.21	1.16
**kdgA**	Bifunctional 4-hydroxy-2-oxoglutarate aldolase/2-dehydro-3-deoxy-phosphogluconate aldolase	WP_000800494.1	22 kDa	0.00095	0.21	1.16
**grxC**	Glutaredoxin 3	WP_001273795.1	9 kDa	0.001	0.21	1.16
**pncB**	Nicotinate phosphoribosyltransferase	WP_000191399.1	46 kDa	0.0055	0.21	1.16
**SDR_a4**	SDR family oxidoreductase	WP_099800481.1	30 kDa	0.0056	0.21	1.16
**ytfP**	Gamma-glutamylcyclotransferase	WP_001219167.1	13 kDa	0.018	0.21	1.16
**PRK10562**	N-acetyltransferase	WP_079960948.1	17 kDa	0.019	0.21	1.16
**DUR1B**	Biotin-dependent carboxyltransferase family protein	WP_000932038.1	34 kDa	0.00017	0.2	1.15
**hflC**	Protease modulator HflC	WP_080201863.1	34 kDa	0.0004	0.2	1.15
**murE**	UDP-N-acetylmuramoyl-L-alanyl-D-glutamate--2,6-diaminopimelate ligase	WP_000775071.1	53 kDa	0.001	0.2	1.15
**pfkB**	6-phosphofructokinase II	WP_000251697.1	33 kDa	0.002	0.2	1.15
**mukF**	Chromosome partition protein MukF	WP_061449547.1	50 kDa	0.003	0.2	1.15
**gpmM**	2,3-bisphosphoglycerate-independent phosphoglycerate mutase	WP_000116576.1	56 kDa	0.0051	0.2	1.15
**yejK**	Nucleoid-associated protein YejK	WP_000050807.1	38 kDa	0.007	0.2	1.15
**feoB**	Fe(2+) transporter permease subunit FeoB	WP_000736978.1	84 kDa	0.015	0.2	1.15
**accB**	Acetyl-CoA carboxylase biotin carboxyl carrier protein	WP_000354626.1	17 kDa	0.016	0.2	1.15
**stpA**	DNA-binding protein StpA	WP_079960897.1	16 kDa	0.018	0.2	1.15
**tal**	Transaldolase	WP_000130175.1	35 kDa	0.02	0.2	1.15
**wecC**	UDP-N-acetyl-D-mannosamine dehydrogenase	WP_000011236.1	45 kDa	0.001	0.19	1.14
**recB**	Exodeoxyribonuclease V subunit beta	WP_023232686.1	134 kDa	0.002	0.19	1.14
**dapF**	Diaminopimelate epimerase	WP_001160671.1	30 kDa	0.005	0.19	1.14
**ahpF**	Alkyl hydroperoxide reductase subunit F	WP_000887641.1	56 kDa	0.006	0.19	1.14
**alaC**	Alanine transaminase	WP_016716124.1	46 kDa	0.0069	0.19	1.14
**rdgC**	Recombination-associated protein RdgC	WP_000964305.1	34 kDa	0.01	0.19	1.14
**sppA**	Signal peptide peptidase SppA	WP_079959242.1	67 kDa	0.011	0.19	1.14
**ligA**	NAD-dependent DNA ligase LigA	WP_000433288.1	73 kDa	0.00059	0.18	1.13
**mutL**	DNA mismatch repair endonuclease MutL	WP_001122556.1	68 kDa	0.004	0.18	1.13
**nlpD**	Peptidoglycan DD-metalloendopeptidase family protein	WP_000244318.1	27 kDa	0.0042	0.18	1.13
**dnaJ**	Molecular chaperone DnaJ	WP_001119009.1	41 kDa	0.011	0.18	1.13
**gpmA**	2,3-diphosphoglycerate-dependent phosphoglycerate mutase	WP_000301554.1	28 kDa	0.011	0.18	1.13
**rodZ**	Cytoskeleton protein RodZ	WP_001090883.1	36 kDa	0.016	0.18	1.13
**rbsB**	Ribose ABC transporter substrate-binding protein RbsB	WP_023254734.1	31 kDa	0.001	0.17	1.13

Gene / locus name	Protein Function	Accession Number	Molecular Weight	Permutation Test (p-value) Benjamini-Hochberg (p < 0.02370)	Log2 Fold Change	Fold Change
**hemC**	Hydroxymethylbilane synthase	WP_001521319.1	34 kDa	0.002	0.17	1.13
**hflX**	GTPase HflX	WP_000460338.1	48 kDa	0.003	0.17	1.13
**rapA**	RNA polymerase-associated protein RapA	WP_001116966.1	110 kDa	0.0037	0.17	1.13
**yidC**	Membrane protein insertase YidC	WP_000378272.1	62 kDa	0.0046	0.17	1.13
**rnpA**	Ribonuclease P protein component	WP_000239725.1	14 kDa	0.005	0.17	1.13
**alaA**	Pyridoxal phosphate-dependent aminotransferase	WP_000074540.1	45 kDa	0.0059	0.17	1.13
**uvrD**	DNA helicase II	WP_076927197.1	82 kDa	0.00013	0.16	1.12
**crp**	cAMP-activated global transcriptional regulator CRP	WP_000242746.1	24 kDa	0.00033	0.16	1.12
**hslU**	HslU--HslV peptidase ATPase subunit	WP_001293355.1	50 kDa	0.001	0.16	1.12
**tyrR**	Transcriptional regulator TyrR	WP_001235489.1	58 kDa	0.002	0.16	1.12
**plsB**	Glycerol-3-phosphate 1-O-acyltransferase PlsB	WP_000017360.1	91 kDa	0.0031	0.16	1.12
**yaaA**	Peroxide stress protein YaaA	WP_000906176.1	30 kDa	0.004	0.16	1.12
**ribE**	6,7-dimethyl-8-ribityllumazine synthase	WP_001021372.1	16 kDa	0.005	0.16	1.12
**damX**	Cell division protein DamX	WP_000343146.1	45 kDa	0.011	0.16	1.12
**murI**	Glutamate racemase	WP_031619622.1	31 kDa	0.013	0.16	1.12
**pepQ**	Xaa-Pro dipeptidase	WP_000444527.1	50 kDa	0.001	0.15	1.11
**artJ**	Arginine ABC transporter substrate-binding protein	WP_000756583.1	27 kDa	0.002	0.15	1.11
**rnc**	Ribonuclease III	WP_001068341.1	26 kDa	0.006	0.15	1.11
**rph**	Ribonuclease PH	WP_065619007.1	25 kDa	0.01	0.15	1.11
**allR**	HTH-type transcriptional repressor AllR	WP_000141265.1	29 kDa	0.013	0.15	1.11
**mutS**	DNA mismatch repair protein MutS	WP_046340808.1	95 kDa	0.021	0.15	1.11
**dacB**	Serine-type D-Ala-D-Ala carboxypeptidase	WP_001212675.1	52 kDa	0.002	0.14	1.10
**ispG**	Flavodoxin-dependent (E)-4-hydroxy-3-methylbut-2-enyl-diphosphate synthase	WP_000551804.1	41 kDa	0.005	0.14	1.10
**dnaG**	DNA primase	WP_000918865.1	65 kDa	0.0067	0.14	1.10
**arnA**	Bifunctional UDP-4-amino-4-deoxy-L-arabinose formyltransferase/UDP-glucuronic acid oxidase ArnA	WP_038425381.1	73 kDa	0.02	0.12	1.09
**groL**	Chaperonin GroEL	WP_000729126.1	57 kDa	0.017	0.1	1.07
**purB**	Adenylosuccinate lyase	WP_000423763.1	52 kDa	0.014	-0.09	0.94
**rarA**	Replication-associated recombination protein A	WP_000067792.1	50 kDa	0.019	-0.11	0.93
**pflB**	Formate C-acetyltransferase	WP_001292799.1	85 kDa	0.004	-0.12	0.92
**rplE**	50S ribosomal protein L5	WP_001096206.1	20 kDa	0.02	-0.12	0.92
**glmU**	Bifunctional UDP-N-acetylglucosamine diphosphorylase/glucosamine-1-phosphate N-acetyltransferase GlmU	WP_000934854.1	49 kDa	0.0069	-0.13	0.91
**zapB**	Septal ring assembly protein ZapB	WP_000051370.1	9 kDa	0.009	-0.13	0.91
**rpoA**	DNA-directed RNA polymerase subunit alpha	WP_001162094.1	37 kDa	0.001	-0.14	0.91
**galF**	GalU regulator GalF	WP_000981469.1	33 kDa	0.002	-0.14	0.91
**mog**	Molybdopterin adenylyltransferase	WP_000380372.1	21 kDa	0.012	-0.14	0.91
**dsdA**	D-serine ammonia-lyase	WP_000427995.1	47 kDa	0.016	-0.14	0.91
**alr**	Alanine racemase	WP_001147296.1	39 kDa	0.0016	-0.15	0.90
**mgtA**	Magnesium-translocating P-type ATPase	WP_023227692.1	100 kDa	0.002	-0.15	0.90
**slyD**	Peptidylprolyl isomerase	WP_000861341.1	21 kDa	0.004	-0.15	0.90
**nrdB**	Ribonucleoside-diphosphate reductase 1 subunit beta	WP_000332026.1	44 kDa	0.008	-0.15	0.90

Gene / locus name	Protein Function	Accession Number	Molecular Weight	Permutation Test (p-value) Benjamini-Hochberg (p < 0.02370)	Log2 Fold Change	Fold Change
**rplL**	50S ribosomal protein L7/L12	WP_000028882.1	12 kDa	0.02	-0.15	0.90
**maeB**	NADP-dependent oxaloacetate-decarboxylating malate dehydrogenase	WP_000344299.1	82 kDa	0.00059	-0.16	0.90
**rpsA**	30S ribosomal protein S1	WP_000140324.1	61 kDa	0.001	-0.16	0.90
**rpsN**	30S ribosomal protein S14	WP_001118932.1	12 kDa	0.011	-0.16	0.90
**rmsG**	16S rRNA (guanine(527)-N(7))-methyltransferase RsmG	WP_001519938.1	23 kDa	0.012	-0.16	0.90
**ompR**	Two-component system response regulator OmpR	WP_001157751.1	27 kDa	0.021	-0.16	0.90
	Hypothetical protein	WP_001727159.1	45 kDa	0.005	-0.16	0.90
**rpsI**	30S ribosomal protein S9	WP_000829815.1	15 kDa	0.001	-0.17	0.89
**helD**	DNA helicase IV	WP_138624231.1	78 kDa	0.002	-0.17	0.89
**DUF3748**	DUF3748 domain-containing protein	WP_000809945.1	45 kDa	0.023	-0.17	0.89
**rpsP**	30S ribosomal protein S16	WP_000256453.1	9 kDa	0.001	-0.18	0.88
**mlaA**	Phospholipid-binding lipoprotein MlaA	WP_000776787.1	28 kDa	0.012	-0.18	0.88
**tbpA**	Thiamine ABC transporter substrate binding subunit	WP_000915326.1	36 kDa	0.00083	-0.19	0.88
**infC**	Translation initiation factor IF-3	WP_038390121.1	21 kDa	0.002	-0.19	0.88
**rluC**	23S rRNA pseudouridine(955/2504/2580) synthase RluC	WP_000846319.1	36 kDa	0.011	-0.19	0.88
**ygiN**	Antibiotic biosynthesis monooxygenase	WP_000958587.1	12 kDa	0.011	-0.19	0.88
**purE**	5-(carboxyamino)imidazole ribonucleotide mutase	WP_000098736.1	18 kDa	0.016	-0.19	0.88
**prmA**	50S ribosomal protein L11 methyltransferase	WP_001145849.1	32 kDa	0.001	-0.2	0.87
**pgeF**	Polyphenol oxidase	WP_000992636.1	26 kDa	0.003	-0.2	0.87
**udp**	Uridine phosphorylase	WP_000045169.1	27 kDa	0.01	-0.2	0.87
**lysR**	LysR family transcriptional regulator	WP_135416390.1	34 kDa	0.012	-0.2	0.87
**crr**	PTS glucose transporter subunit IIA	WP_000522253.1	18 kDa	0.014	-0.2	0.87
**rpoZ**	DNA-directed RNA polymerase subunit omega	WP_000135058.1	10 kDa	0.021	-0.2	0.87
	PTS sugar transporter subunit IIC	WP_058222797.1	47 kDa	0.007	-0.2	0.87
**rplA**	50S ribosomal protein L1	WP_001096676.1	25 kDa	0.0025	-0.21	0.86
**fliM**	Flagellar motor switch protein FliM	WP_000502811.1	38 kDa	0.004	-0.21	0.86
**rlmB**	23S rRNA (guanosine(2251)-2'-O)-methyltransferase RlmB	WP_001293274.1	27 kDa	0.0056	-0.21	0.86
**cobT**	Nicotinate-nucleotide--dimethylbenzimidazole phosphoribosyltransferase	WP_001193968.1	37 kDa	0.006	-0.21	0.86
**rlmG**	23S rRNA (guanine(1835)-N(2))-methyltransferase RlmG	WP_000019983.1	42 kDa	0.008	-0.21	0.86
**yrfG**	GMP/IMP nucleotidase	WP_001520508.1	25 kDa	0.017	-0.21	0.86
**glmM**	Phosphoglucosamine mutase	WP_000071169.1	47 kDa	0.001	-0.22	0.86
**phoU**	Phosphate signaling complex protein PhoU	WP_000377800.1	27 kDa	0.001	-0.22	0.86
**rlmA**	23S rRNA (guanine(745)-N(1))-methyltransferase	WP_000010916.1	30 kDa	0.001	-0.22	0.86
**ybcJ**	Ribosome-associated protein YbcJ	WP_000190281.1	7 kDa	0.004	-0.22	0.86
**glpX**	Class II fructose-bisphosphatase	WP_001250616.1	36 kDa	0.009	-0.22	0.86
**glxK**	Glycerate kinase	WP_000706477.1	39 kDa	0.012	-0.22	0.86
**corA**	Magnesium/cobalt transporter CorA	WP_000947139.1	37 kDa	0.014	-0.22	0.86
**speE**	Polyamine aminopropyltransferase	WP_000829968.1	32 kDa	0.016	-0.22	0.86
**yaiL**	DUF2058 domain-containing protein	WP_001096724.1	20 kDa	0.004	-0.23	0.85
**panM**	Aspartate 1-decarboxylase autocleavage activator PanM	WP_135423725.1	14 kDa	0.008	-0.23	0.85

Gene / locus name	Protein Function	Accession Number	Molecular Weight	Permutation Test (p-value) Benjamini-Hochberg (p < 0.02370)	Log2 Fold Change	Fold Change
**acpP**	Acyl carrier protein	WP_000103754.1	9 kDa	0.0013	-0.24	0.85
**yjaG**	DUF416 family protein	WP_000940092.1	23 kDa	0.003	-0.24	0.85
**uspE**	Universal stress protein UspE	WP_001262138.1	36 kDa	0.005	-0.24	0.85
**pflA**	Pyruvate formate lyase 1-activating protein	WP_000067973.1	30 kDa	0.006	-0.24	0.85
**ruvB**	Holliday junction branch migration DNA helicase RuvB	WP_000568508.1	37 kDa	0.012	-0.24	0.85
**pyrE**	Orotate phosphoribosyltransferase	WP_000806169.1	24 kDa	0.013	-0.24	0.85
**ppa**	Inorganic diphosphatase	WP_000055079.1	20 kDa	0.021	-0.24	0.85
**hybO**	Hydrogenase 2 small subunit	WP_000145428.1	40 kDa	0.001	-0.25	0.84
**nudF**	ADP-ribose diphosphatase	WP_001231950.1	24 kDa	0.002	-0.25	0.84
**yhaK**	Pirin family protein	WP_000639789.1	26 kDa	0.021	-0.25	0.84
**flhA**	Flagellar biosynthesis protein FlhA	WP_000002386.1	75 kDa	0.0006	-0.26	0.84
**AKR_SF**	NADP(H)-dependent aldo-keto reductase	WP_146639937.1	33 kDa	0.006	-0.26	0.84
**lsrF**	3-hydroxy-5-phosphonooxypentane-2,4-dione thiolase	WP_000774143.1	32 kDa	0.008	-0.26	0.84
**norR**	Nitric oxide reductase transcriptional regulator NorR	WP_065625151.1	55 kDa	0.003	-0.27	0.83
**purF**	Amidophosphoribosyltransferase	WP_000334204.1	57 kDa	0.006	-0.27	0.83
**pstB**	Phosphate ABC transporter ATP-binding protein PstB	WP_000063118.1	29 kDa	0.00017	-0.28	0.82
**nfsA**	Nitroreductase NfsA	WP_000075300.1	27 kDa	0.00072	-0.28	0.82
**panD**	Aspartate 1-decarboxylase	WP_000621525.1	14 kDa	0.00095	-0.28	0.82
**rpsH**	30S ribosomal protein S8	WP_000062611.1	14 kDa	0.001	-0.28	0.82
**pyrF**	Orotidine-5'-phosphate decarboxylase	WP_023230981.1	26 kDa	0.012	-0.28	0.82
**flhE**	Flagellar protein FlhE	WP_001233619.1	14 kDa	0.0019	-0.3	0.81
**amiA**	N-acetylmuramoyl-L-alanine amidase AmiA	WP_069040808.1	32 kDa	0.002	-0.3	0.81
**btuE**	Glutathione peroxidase	WP_001181565.1	20 kDa	0.003	-0.3	0.81
**aas**	Bifunctional acyl-ACP--phospholipid O-acyltransferase/long-chain-fatty-acid--ACP ligase	WP_023890977.1	80 kDa	0.00095	-0.31	0.81
**fadR**	FadR family transcriptional regulator	WP_000388417.1	30 kDa	0.006	-0.31	0.81
**holE**	DNA polymerase III subunit theta	WP_000856224.1	9 kDa	0.011	-0.31	0.81
**ugpC**	Sn-glycerol-3-phosphate import ATP-binding protein UgpC	WP_000907837.1	39 kDa	0.004	-0.32	0.80
**smrB**	Endonuclease SmrB	WP_058222767.1	21 kDa	0.005	-0.32	0.80
	Aryl-sulfate sulfotransferase	WP_069057620.1	67 kDa	0.005	-0.32	0.80
**entC**	Isochorismate synthase EntC	WP_000367597.1	43 kDa	0.02	-0.32	0.80
**thiG**	Thiazole synthase	WP_000944068.1	27 kDa	0.002	-0.33	0.80
**yccS**	Membrane protein	WP_000269315.1	79 kDa	0.012	-0.33	0.80
	Hypothetical protein	WP_000936453.1	15 kDa	0.002	-0.33	0.80
**mlaB**	Lipid asymmetry maintenance protein MlaB	WP_000188843.1	11 kDa	0.018	-0.34	0.79
**ptsG**	PTS glucose transporter subunit IIBC	WP_000475705.1	50 kDa	0.001	-0.35	0.78
**PRK00711**	D-amino acid dehydrogenase	WP_001266937.1	48 kDa	0.017	-0.35	0.78
	Mechanosensitive ion channel family protein	WP_001156965.1	47 kDa	0.015	-0.35	0.78
**nrfA**	Ammonia-forming nitrite reductase cytochrome c552 subunit	WP_061104304.1	54 kDa	0.001	-0.36	0.78
**virK**	VirK family antimicrobial peptide resistance protein	WP_000178732.1	36 kDa	0.001	-0.38	0.77
**wrbA**	NAD(P)H:quinone oxidoreductase	WP_001062894.1	21 kDa	0.001	-0.38	0.77

Gene / locus name	Protein Function	Accession Number	Molecular Weight	Permutation Test (p-value) Benjamini-Hochberg (p < 0.02370)	Log2 Fold Change	Fold Change
**ilvY**	HTH-type transcriptional activator IlvY	WP_000365798.1	33 kDa	0.001	-0.38	0.77
**ycaR**	Protein YcaR	WP_000350061.1	7 kDa	0.004	-0.38	0.77
**rbsD**	D-ribose pyranase	WP_000715944.1	15 kDa	0.006	-0.38	0.77
**dmsA**	Dimethyl sulfoxide reductase subunit A	WP_079958286.1	90 kDa	0.004	-0.39	0.76
**fabD**	ACP S-malonyltransferase	WP_000191342.1	32 kDa	0.005	-0.39	0.76
**nrdD**	Anaerobic ribonucleoside-triphosphate reductase	WP_000187818.1	80 kDa	0.002	-0.4	0.76
**ptsH**	Phosphocarrier protein Hpr	WP_000487600.1	9 kDa	0.002	-0.4	0.76
**pspB**	Envelope stress response membrane protein PspB	WP_001274953.1	9 kDa	0.018	-0.4	0.76
**DUF1116**	DUF1116 domain-containing protein	WP_000495333.1	45 kDa	0.004	-0.41	0.75
**pheP**	Phenylalanine transporter	WP_000786283.1	51 kDa	0.02	-0.41	0.75
**rfbA**	Glucose-1-phosphate thymidylyltransferase RfbA	WP_000857529.1	32 kDa	0.00039	-0.42	0.75
**tdk**	Thymidine kinase	WP_000068097.1	23 kDa	0.001	-0.43	0.74
**mtlD**	Mannitol dehydrogenase family protein	WP_001181255.1	54 kDa	0.001	-0.43	0.74
**rsmI**	16S rRNA (cytidine(1402)-2'-O)-methyltransferase	WP_001751326.1	32 kDa	0.002	-0.43	0.74
**osmE**	Osmotically-inducible lipoprotein OsmE	WP_001039301.1	12 kDa	0.003	-0.43	0.74
**acrE**	Efflux RND transporter periplasmic adaptor subunit	WP_000160380.1	41 kDa	0.02	-0.43	0.74
**yjiA**	GTPase	WP_000187839.1	36 kDa	0.00013	-0.44	0.74
**tsx**	Nucleoside-specific channel-forming protein Tsx	WP_000752021.1	33 kDa	0.004	-0.44	0.74
**lsrB**	Autoinducer 2 ABC transporter substrate-binding protein LsrB	WP_079920817.1	37 kDa	0.002	-0.45	0.73
**gntR**	GntR family transcriptional regulator	WP_023227759.1	25 kDa	0.002	-0.45	0.73
**pmrG**	Lipopolysaccharide core heptose(II)-phosphate phosphatase	WP_000879258.1	22 kDa	0.003	-0.45	0.73
**feoA**	Ferrous iron transporter A	WP_061451033.1	8 kDa	0.01	-0.45	0.73
**rsuA**	16S rRNA pseudouridine synthase RsuA	WP_001234834.1	26 kDa	0.001	-0.46	0.73
**PRK10458**	DNA cytosine methyltransferase	WP_001157304.1	54 kDa	0.00016	-0.47	0.72
**PHA03247**	Hypothetical protein	WP_001129953.1	47 kDa	0.001	-0.47	0.72
**yecR**	Lipoprotein	WP_000803230.1	12 kDa	0.002	-0.47	0.72
**ldtD**	L,D-transpeptidase	WP_000925899.1	68 kDa	0.002	-0.48	0.72
**hemG**	Menaquinone-dependent protoporphyrinogen IX dehydrogenase	WP_000853952.1	21 kDa	0.0021	-0.48	0.72
**prtC**	U32 family peptidase	WP_000421309.1	37 kDa	0.003	-0.49	0.71
**fumD**	Fumarate hydratase FumD	WP_001727516.1	8 kDa	0.007	-0.49	0.71
**tilS**	tRNA lysidine synthetase TilS	WP_080202188.1	48 kDa	0.001	-0.5	0.71
**solA**	N-methyl-L-tryptophan oxidase	WP_000872763.1	41 kDa	0.00063	-0.51	0.70
**dusB**	tRNA dihydrouridine synthase DusB	WP_001219664.1	36 kDa	0.017	-0.51	0.70
**rimO**	30S ribosomal protein S12 methylthiotransferase RimO	WP_000073317.1	50 kDa	0.023	-0.51	0.70
**invH**	Invasion protein	WP_001529477.1	14 kDa	0.002	-0.52	0.70
**PRK10314**	GNAT family N-acetyltransferase	WP_000568157.1	17 kDa	0.0027	-0.52	0.70
**PRK11561**	Isovaleryl-CoA dehydrogenase	WP_000118960.1	60 kDa	0.013	-0.52	0.70
**dusA**	tRNA dihydrouridine synthase DusA	WP_048349013.1	39 kDa	0.00012	-0.53	0.69
**hscC**	Molecular chaperone HscC	WP_000368024.1	62 kDa	0.002	-0.53	0.69
**fliL**	Flagellar basal body-associated protein FliL	WP_000132169.1	17 kDa	0.001	-0.54	0.69

Gene / locus name	Protein Function	Accession Number	Molecular Weight	Permutation Test (p-value) Benjamini-Hochberg ($p < 0.02370$)	Log2 Fold Change	Fold Change
**flgD**	Flagellar hook assembly protein FlgD	WP_000020450.1	24 kDa	0.002	-0.54	0.69
**mutT**	8-oxo-dGTP diphosphatase MutT	WP_000736055.1	15 kDa	0.00057	-0.55	0.68
**yedE**	Selenium metabolism membrane protein YedE/FdhT	WP_000580669.1	46 kDa	0.002	-0.55	0.68
**marA**	MDR efflux pump AcrAB transcriptional activator MarA	WP_011233048.1	17 kDa	0.004	-0.55	0.68
**cheZ**	Protein phosphatase CheZ	WP_000983585.1	24 kDa	0.001	-0.56	0.68
**gluQ**	tRNA glutamyl-Q(34) synthetase GluQRS	WP_001682576.1	34 kDa	0.02	-0.56	0.68
**rluF**	23S rRNA pseudouridine synthase RluF	WP_000954610.1	32 kDa	0.001	-0.58	0.67
**opgE**	Phosphoethanolamine transferase	WP_001089326.1	60 kDa	0.001	-0.58	0.67
	HAD family hydrolase	WP_076933647.1	30 kDa	0.003	-0.61	0.66
**yacL**	UPF0231 family protein	WP_000384308.1	14 kDa	0.004	-0.61	0.66
**flgK**	Flagellar hook-associated protein FlgK	WP_000096422.1	59 kDa	0.00029	-0.62	0.65
**yicI**	Alpha-xylosidase	WP_000702987.1	88 kDa	0.009	-0.62	0.65
**PDC1**	Alpha-keto acid decarboxylase family protein	WP_038394309.1	60 kDa	0.002	-0.63	0.65
**GnsAB_toxin**	Addiction module toxin, GnsA/GnsB family	WP_001083582.1	6 kDa	0.002	-0.63	0.65
**pagN**	Adhesin/invasin protein PagN	WP_000787603.1	26 kDa	0.004	-0.64	0.64
**gcd**	Pyrroloquinoline quinone-dependent dehydrogenase	WP_001726933.1	86 kDa	0.0031	-0.66	0.63
	Hypothetical protein	WP_000185028.1	60 kDa	0.02	-0.66	0.63
	Hypothetical protein	WP_001725572.1	16 kDa	0.01	-0.67	0.63
**PRK06184**	FAD-dependent oxidoreductase	WP_000199421.1	56 kDa	0.002	-0.68	0.62
**ccmH**	Cytochrome c-type biogenesis protein CcmH	WP_038394509.1	40 kDa	0.014	-0.68	0.62
**patD**	Aminobutyraldehyde dehydrogenase	WP_000158087.1	52 kDa	0.004	-0.7	0.62
**modC**	Molybdenum ABC transporter ATP-binding protein ModC	WP_000891715.1	39 kDa	0.00037	-0.71	0.61
**ycgR**	Flagellar brake protein YcgR	WP_079898488.1	28 kDa	0.001	-0.72	0.61
**ccmE**	Cytochrome c maturation protein CcmE	WP_001053600.1	18 kDa	0.011	-0.72	0.61
**gfa**	Hypothetical protein	WP_001700938.1	10 kDa	0.002	-0.74	0.60
**PRK12318**	Hypothetical protein	WP_000248794.1	45 kDa	0.004	-0.74	0.60
**spaK**	SPI-1 type III secretion system chaperone SpaK	WP_001164066.1	15 kDa	0.004	-0.77	0.59
**DUF3300**	DUF3300 domain-containing protein	WP_023891266.1	59 kDa	0.006	-0.77	0.59
**ansP**	L-asparagine permease	WP_000857110.1	54 kDa	0.0015	-0.78	0.58
**trmN**	tRNA(1)(Val) (adenine(37)-N(6))-methyltransferase TrmN	WP_000083339.1	27 kDa	0.007	-0.78	0.58
**gatZ**	Tagatose-bisphosphate aldolase subunit GatZ	WP_000658736.1	47 kDa	0.001	-0.79	0.58
**folA**	Type 3 dihydrofolate reductase	WP_000624379.1	18 kDa	0.002	-0.79	0.58
**srlE**	PTS glucitol/sorbitol transporter subunit IIB	WP_000199033.1	34 kDa	0.007	-0.81	0.57
**add**	Adenosine deaminase	WP_000565565.1	36 kDa	0.00016	-0.82	0.57
**steA**	Hypothetical protein	WP_001120854.1	24 kDa	0.0037	-0.84	0.56
**allD**	Ureidoglycolate dehydrogenase	WP_000703934.1	38 kDa	0.004	-0.84	0.56
	NUDIX hydrolase	WP_000476070.1	17 kDa	0.015	-0.84	0.56
**narK**	Nitrate transporter NarK	WP_000019850.1	50 kDa	0.001	-0.85	0.55
**argF**	Ornithine carbamoyltransferase	WP_000103033.1	37 kDa	0.002	-0.86	0.55
**adhP**	NAD(P)-dependent alcohol dehydrogenase	WP_001727706.1	36 kDa	0.004	-0.86	0.55

Gene / locus name	Protein Function	Accession Number	Molecular Weight	Permutation Test (p-value) Benjamini-Hochberg (p < 0.02370)	Log2 Fold Change	Fold Change
**lrhA**	Transcriptional regulator LrhA	WP_000606287.1	35 kDa	0.004	-0.87	0.55
**mtfA**	DgsA anti-repressor MtfA	WP_000598921.1	30 kDa	0.004	-0.88	0.54
**hypT**	Hypochlorite stress DNA-binding transcriptional regulator HypT	WP_080165157.1	35 kDa	0.004	-0.88	0.54
**flgA**	Flagellar basal body P-ring formation protein FlgA	WP_001194076.1	24 kDa	0.002	-0.89	0.54
**lipo_YgdI_YgdR**	YgdI/YgdR family lipoprotein	WP_000750393.1	8 kDa	0.004	-0.93	0.52
**glgB**	1,4-alpha-glucan branching enzyme	WP_000098543.1	84 kDa	0.00021	-0.95	0.52
**fepC**	Iron-enterobactin ABC transporter ATP-binding protein	WP_023227378.1	29 kDa	0.004	-0.96	0.51
**orgB**	Oxygen-regulated invasion protein OrgB	WP_058222372.1	26 kDa	0.002	-0.97	0.51
**fimZ**	Fimbria biosynthesis transcriptional regulator FimZ	WP_079901676.1	24 kDa	0.004	-0.97	0.51
**emrD**	Multidrug efflux MFS transporter EmrD	WP_000828735.1	42 kDa	0.007	-0.98	0.51
**ompC**	Porin OmpC	WP_000758335.1	41 kDa	0.002	-1.01	0.50
	Mechanosensitive ion channel family protein	WP_061451022.1	43 kDa	0.004	-1.02	0.49
**torD**	Molecular chaperone	WP_001112216.1	25 kDa	0.004	-1.02	0.49
**citE**	Citrate (pro-3S)-lyase subunit beta	WP_050946835.1	33 kDa	0.001	-1.06	0.48
**livK**	Branched-chain amino acid ABC transporter substrate-binding protein	WP_000676957.1	39 kDa	0.003	-1.06	0.48
**cheW**	Chemotaxis protein CheW	WP_000147295.1	18 kDa	0.002	-1.07	0.48
**phsB**	Thiosulfate reductase electron transport protein PhsB	WP_001015351.1	21 kDa	0.003	-1.08	0.47
**cutC**	Copper homeostasis protein CutC	WP_001185769.1	27 kDa	0.00048	-1.09	0.47
**sptP**	SPI-1 type III secretion system effector GTPase-activating protein SptP	WP_023227558.1	60 kDa	0.004	-1.09	0.47
**chaB**	Putative cation transport regulator ChaB	WP_001146392.1	9 kDa	0.004	-1.11	0.46
**tusD**	Sulfurtransferase complex subunit TusD	WP_001268010.1	14 kDa	0.004	-1.11	0.46
**ampH**	D-alanyl-D-alanine-carboxypeptidase/endopeptidase AmpH	WP_000830784.1	42 kDa	0.004	-1.13	0.46
**prgK**	Type III secretion system inner membrane ring lipoprotein PrgK	WP_000621238.1	28 kDa	0.004	-1.14	0.45
**pspE**	Thiosulfate sulfurtransferase PspE	WP_000913428.1	12 kDa	0.0013	-1.15	0.45
**lpxR**	Lipid A deacylase LpxR family protein	WP_001046434.1	35 kDa	0.002	-1.17	0.44
**fabG**	SDR family oxidoreductase	WP_000032693.1	27 kDa	0.002	-1.21	0.43
**fdnI**	Formate dehydrogenase N subunit gamma	WP_079959237.1	26 kDa	0.004	-1.23	0.43
**opuC**	ABC transporter substrate-binding protein	WP_000155871.1	33 kDa	0.002	-1.32	0.40
**tctC**	Tripartite tricarboxylate transporter substrate binding protein	WP_000744418.1	30 kDa	0.002	-1.33	0.40
**fimC**	Molecular chaperone FimC	WP_000935025.1	25 kDa	0.004	-1.33	0.40
**dacD**	Serine-type D-Ala-D-Ala carboxypeptidase DacD	WP_000925044.1	43 kDa	0.002	-1.34	0.40
	SAM-dependent methyltransferase	WP_000979781.1	61 kDa	0.004	-1.35	0.39
**pduJ**	Propanediol utilization microcompartment protein PduJ	WP_001057755.1	9 kDa	0.002	-1.44	0.37
**DUF1889**	DUF1889 family protein	WP_001539227.1	12 kDa	0.002	-1.44	0.37
**ompN**	Porin OmpN	WP_000824321.1	42 kDa	0.019	-1.51	0.35
**siiC**	TolC family protein	WP_001747443.1	51 kDa	0.002	-1.56	0.34
**fljA**	Phase 1 flagellin gene repressor FljA	WP_000389002.1	20 kDa	0.004	-1.57	0.34
**pduB**	Propanediol utilization microcompartment protein PduB	WP_001734087.1	21 kDa	0.002	-1.63	0.32
	CDP-abequose synthase	WP_000143399.1	34 kDa	0.002	-1.73	0.30
**#NAME?**	AAA family ATPase	WP_058815804.1	40 kDa	0.00011	-1.76	0.30

Gene / locus name	Protein Function	Accession Number	Molecular Weight	Permutation Test (p-value) Benjamini-Hochberg (p < 0.02370)	Log2 Fold Change	Fold Change
**rop**	Rop family plasmid primer RNA-binding protein	WP_000165985.1	7 kDa	0.0016	-1.78	0.29
**yhbO**	Protein/nucleic acid deglycase	WP_000037599.1	19 kDa	0.004	-1.78	0.29
	Membrane protein	WP_001240360.1	39 kDa	0.002	-1.79	0.29
**PTS_IIA_fru**	PTS sugar transporter subunit IIA	WP_001683480.1	17 kDa	0.004	-1.8	0.29
**sopD2**	Type III secretion system effector SopD2	WP_001145570.1	38 kDa	0.004	-1.82	0.28
**pyrI**	Aspartate carbamoyltransferase regulatory subunit	WP_000148567.1	17 kDa	0.00015	-1.83	0.28
**yodD**	YodD family peroxide/acid resistance protein	WP_000844798.1	9 kDa	0.004	-1.9	0.27
**hemX**	Uroporphyrinogen-III C-methyltransferase	WP_000138954.1	42 kDa	0.002	-1.93	0.26
**invA**	Type III secretion system export apparatus protein InvA	WP_000927212.1	76 kDa	0.002	-1.94	0.26
**sodC**	Superoxide dismutase [Cu-Zn] SodC2	WP_000826818.1	18 kDa	0.002	-1.94	0.26
**glpT**	Glycerol-3-phosphate transporter	WP_080195040.1	44 kDa	0.004	-1.99	0.25
**deoR**	Hypothetical protein	WP_001123709.1	35 kDa	0.001	-2	0.25
**mngR**	GntR family transcriptional regulator	WP_000854956.1	28 kDa	0.004	-2.02	0.25
**sopB**	SPI-1 type III secretion system effector inositol phosphate phosphatase SopB	WP_001166955.1	62 kDa	0.009	-2.02	0.25
**pduE**	Propanediol dehydratase small subunit PduE	WP_001090597.1	19 kDa	0.004	-2.06	0.24
**dnaE**	DNA polymerase III subunit alpha	WP_001294818.1	130 kDa	0.004	-2.08	0.24
**kduD**	2-dehydro-3-deoxy-D-gluconate 5-dehydrogenase KduD	WP_000602485.1	27 kDa	0.004	-2.25	0.21
**DUF3617**	DUF3617 domain-containing protein	WP_000826195.1	18 kDa	0.002	-2.31	0.20
**dinI**	DinI family protein	WP_001217665.1	9 kDa	0.004	-2.36	0.19
**gatB**	PTS sugar transporter subunit IIB	WP_000723161.1	10 kDa	0.004	-2.41	0.19
**Zn Tnp IS91**	Replication protein	WP_100793785.1	35 kDa	0.002	-2.42	0.19
**suhB**	Inositol-1-monophosphatase	WP_000553467.1	29 kDa	0.004	-2.48	0.18
	Isochorismatase family protein	WP_000106461.1	24 kDa	0.0019	-2.54	0.17
**mbeD**	Mobilization protein MbeD	WP_029401705.1	9 kDa	0.004	-2.61	0.16
**yciE**	Ferritin-like domain-containing protein	WP_001109977.1	19 kDa	0.002	-3.04	0.12
**PRK10977**	YjjI family glycine radical enzyme	WP_001111688.1	58 kDa	0.004	-3.08	0.12
**rfbB**	dTDP-glucose 4,6-dehydratase	WP_000697840.1	41 kDa	0.004	-3.09	0.12
**ilvC**	Ketol-acid reductoisomerase	WP_000024932.1	54 kDa	0.0017	-3.23	0.11
**agaS**	SIS domain-containing protein	WP_023206877.1	37 kDa	0.004	-3.29	0.10
**dmsA_ynfE**	Molybdopterin-dependent oxidoreductase	WP_077917388.1	89 kDa	0.004	-3.39	0.10
	Hypothetical protein	WP_000010697.1	22 kDa	0.00014	-3.75	0.07
**MbeB N**	Mobilization protein	WP_140252074.1	20 kDa	0.002	-4.34	0.05
**valS**	Valine--tRNA ligase	WP_000416322.1	108 kDa	0.002	-5.41	0.02
**nsfB**	Oxygen-insensitive NAD(P)H nitroreductase	WP_000355870.1	24 kDa	0.004	-6.25	0.01
**coaBC**	Bifunctional phosphopantothenoylcysteine decarboxylase/phosphopantothenate--cysteine ligase CoaBC	WP_000050176.1	43 kDa	0.004	-10.89	0.00
