# Supplementary material for: Novel Insight into the Effects of CpxR on Salmonella enteritidis Cells during the Chlorhexidine Treatment and Non-Stressful Growing Conditions
Source: Int J Mol Sci. 2021 Aug 19;22(16):8938. doi: 10.3390/ijms22168938 (PMC8396259; doi:10.3390/ijms22168938)
Supplement: Supplementary file 1 [file ijms-22-08938-s001.zip › Supplementary/supplementary Table S2.pdf]

Gene / locus name	Protein Function	Accession Number	Molecular Weight	Permutation Test (p-value) Benjamini-Hochberg (p < 0.02596)	Log2 Fold Change	Fold Change
**tsr**	Methyl-accepting chemotaxis protein	WP_038425819.1	60 kDa	0.00021	9.69	826.0011614
**WP_001574213**	Hypothetical protein	WP_001574213.1	20 kDa	0.006	5.54	46.52712055
**yjjI**	YjjI family glycine radical enzyme	WP_001111683.1	58 kDa	0.01	4.25	19.02731384
**rfbB**	dTDP-glucose 4,6-dehydratase	WP_000697848.1	41 kDa	0.006	3.87	14.6213032
**WP_001539312**	YajQ family cyclic di-GMP-binding protein	WP_001539312.1	19 kDa	0.021	3.79	13.8325957
**WP_023227560.1**	YebC/PmpR family DNA-binding transcriptional regulator	WP_000907242.1	26 kDa	0.006	3.77	13.64215827
**stbD**	Type II toxin-antitoxin system Phd/YefM family antitoxin	WP_000481003.1	8 kDa	0.006	3.67	12.72858374
**WP_001244219**	DUF2732 family protein	WP_001244219.1	9 kDa	0.012	3.56	11.79415374
**proA**	Glutamate-5-semialdehyde dehydrogenase	WP_000893239.1	45 kDa	0.006	3.14	8.815240927
**WP_000932273**	Phage repressor protein	WP_000932273.1	23 kDa	0.006	3.05	8.282119391
**rpsG**	30S ribosomal protein S7	WP_001138043.1	18 kDa	0.006	3.04	8.224910613
**rne**	Ribonuclease E	WP_079920831.1	119 kDa	0.006	3.02	8.111675838
**clpB**	ATP-dependent chaperone ClpB	WP_024156384.1	95 kDa	0.006	3	8
**tnpR**	Helix-turn-helix domain-containing protein	WP_001705923.1	21 kDa	0.00012	2.97	7.835362381
**WP_000336848**	DUF1496 domain-containing protein	WP_000336848.1	10 kDa	0.006	2.81	7.012845771
**hsdM**	Site-specific DNA-methyltransferase	WP_000910353.1	73 kDa	0.001	2.74	6.680703355
**nemA**	Alkene reductase	WP_000092935.1	40 kDa	0.006	2.49	5.617779503
**WP_001083931**	DUF1481 domain-containing protein	WP_001083931.1	26 kDa	0.006	2.46	5.502167273
**mukB**	Chromosome partition protein MukB	WP_000572746.1	170 kDa	0.0051	2.31	4.9588308
**WP_000863534**	Type I restriction-modification protein specificity subunit	WP_000863534.1	48 kDa	0.006	2.26	4.789914818
**WP_000722370**	DUF2511 domain-containing protein	WP_000722370.1	13 kDa	0.001	2.22	4.658934346
**traM**	Relaxosome protein TraM	WP_079983192.1	14 kDa	0.006	2.15	4.438277888
**plaP**	Putrescine/proton symporter PlaP	WP_000178807.1	50 kDa	0.022	2.12	4.34693945
**yeaD**	D-hexose-6-phosphate mutarotase	WP_000608660.1	32 kDa	0.006	2.05	4.141059695
**relB**	Type II toxin-antitoxin system RelB/DinJ family antitoxin	WP_000729713.1	9 kDa	0.006	2.05	4.141059695
**WP_001240331**	Recombinase family protein	WP_001240331.1	21 kDa	0.001	1.8	3.482202253
**ptsP**	Phosphoenolpyruvate--protein phosphotransferase	WP_079829802.1	85 kDa	0.00038	1.75	3.363585661
**hcp**	Hydroxylamine reductase	WP_000458773.1	60 kDa	0.006	1.45	2.732080514
**hemF**	Oxygen-dependent coproporphyrinogen oxidase	WP_000801332.1	34 kDa	0.006	1.44	2.713208655
**WP_079957070**	Thioredoxin domain-containing protein	WP_079957070.1	24 kDa	0.006	1.43	2.694467154
**prmC**	Peptide chain release factor N(5)-glutamine methyltransferase	WP_000347310.1	31 kDa	0.005	1.33	2.514026749
**WP_000211477**	L-rhamnose isomerase	WP_000211477.1	48 kDa	0.006	1.31	2.4794154
**nfo**	Deoxyribonuclease IV	WP_001729752.1	31 kDa	0.006	1.24	2.361985323
**malS**	Alpha-amylase	WP_000761323.1	76 kDa	0.006	1.17	2.250116969
**ubiF**	Monooxygenase	WP_000047689.1	12 kDa	0.001	1.15	2.219138944
**dinG**	ATP-dependent DNA helicase DinG	WP_001218636.1	81 kDa	0.025	1.08	2.114036081
**hypC**	Hydrogenase 3 maturation protein HypC	WP_022742810.1	10 kDa	0.006	1.05	2.070529848
**gltL**	Glutamate/aspartate ABC transporter ATP binding protein GltL	WP_000631369.1	27 kDa	0.006	1.04	2.056227653
**ccdB**	Type II toxin-antitoxin system toxin CcdB	WP_001159863.1	12 kDa	0.006	0.9	1.866065983
**nirC**	Nitrite transporter NirC	WP_000493575.1	29 kDa	0.012	0.87	1.8276629
**WP_000373611**	Bax inhibitor-1/YccA family protein	WP_000373611.1	26 kDa	0.006	0.86	1.815038311
**pheA**	Bifunctional chorismate mutase/prephenate dehydratase	WP_000200078.1	43 kDa	0.0003	0.81	1.753211443
**coaBC**	Bifunctional phosphopantothenoylcysteine decarboxylase/phosphopantothenate--cysteine ligase CoaBC	WP_099844356.1	59 kDa	0.00068	0.79	1.729074463
**ppiA**	Peptidylprolyl isomerase A	WP_076916862.1	20 kDa	0.006	0.79	1.729074463
**yajL**	Protein deglycase YajL	WP_001275803.1	21 kDa	0.012	0.77	1.705269784
**dnaC**	DNA replication protein DnaC	WP_000799921.1	28 kDa	0.001	0.74	1.670175839
**WP_001034952**	TonB-dependent siderophore receptor	WP_001034952.1	83 kDa	0.0001	0.73	1.658639092
**ispE**	4-(cytidine 5'-diphospho)-2-C-methyl-D-erythritol kinase	WP_000988246.1	31 kDa	0.00054	0.72	1.647182035

Gene / locus name	Protein Function	Accession Number	Molecular Weight	Permutation Test (p-value) Benjamini-Hochberg (p < 0.02596)	Log2 Fold Change	Fold Change
**putP**	Sodium/proline symporter PutP	WP_001018467.1	54 kDa	0.006	0.72	1.647182035
**WP_000436887**	YcbJ family phosphotransferase	WP_000436887.1	34 kDa	0.00015	0.69	1.613283518
**WP_001053173**	YqgE/AlgH family protein	WP_001053173.1	21 kDa	0.002	0.69	1.613283518
**gcl**	Glyoxylate carboligase	WP_001096859.1	65 kDa	0.011	0.69	1.613283518
**yjjP**	Threonine/serine exporter ThrE family protein	WP_001674846.1	28 kDa	0.015	0.68	1.602139755
**malZ**	Maltodextrin glucosidase	WP_080194971.1	68 kDa	0.001	0.67	1.591072968
**dmsA**	Dimethyl sulfoxide reductase subunit A	WP_079958286.1	90 kDa	0.00031	0.66	1.580082624
**glnH2**	Amino acid ABC transporter substrate-binding protein	WP_000588819.1	34 kDa	0.002	0.66	1.580082624
**WP_001708868**	YaiI/YqxD family protein	WP_001708868.1	19 kDa	0.0007	0.65	1.569168196
**hybG**	Hydrogenase maturation factor HybG	WP_000334887.1	9 kDa	0.001	0.65	1.569168196
**ykgJ**	YkgJ family cysteine cluster protein	WP_000114955.1	15 kDa	0.017	0.65	1.569168196
**raiA**	Ribosome-associated translation inhibitor RaiA	WP_000178449.1	13 kDa	0.00035	0.63	1.547564994
**fadR**	GntR family transcriptional regulator	WP_001702649.1	25 kDa	0.005	0.63	1.547564994
**rnd**	Ribonuclease D	WP_001109121.1	42 kDa	0.00054	0.62	1.536875181
**rsmD**	16S rRNA (guanine(966)-N(2))-methyltransferase	WP_000743275.1	22 kDa	0.001	0.62	1.536875181
**WP_080202365**	YggU family protein	WP_080202365.1	8 kDa	0.003	0.62	1.536875181
**aceF**	Pyruvate dehydrogenase complex dihydrolipoyllysine-residue acetyltransferase	WP_000963608.1	66 kDa	0.006	0.62	1.536875181
**leuA**	2-isopropylmalate synthase	WP_000082813.1	57 kDa	0.00011	0.61	1.526259209
**ppnP**	Pyrimidine/purine nucleoside phosphorylase	WP_000941950.1	10 kDa	0.006	0.61	1.526259209
**cyaY**	Iron donor protein CyaY	WP_000999925.1	12 kDa	0.001	0.6	1.515716567
**hypE**	Hydrogenase expression/formation protein HypE	WP_001728890.1	35 kDa	0.004	0.6	1.515716567
**ybgA**	DUF1722 domain-containing protein	WP_138017663.1	36 kDa	0.006	0.6	1.515716567
**ribB**	3,4-dihydroxy-2-butanone-4-phosphate synthase	WP_001076978.1	23 kDa	0.006	0.6	1.515716567
**rpmF**	50S ribosomal protein L32	WP_000290727.1	6 kDa	0.00013	0.58	1.494849249
**cydC**	Cysteine/glutathione ABC transporter ATP-binding protein/permease CydC	WP_001202251.1	63 kDa	0.003	0.58	1.494849249
**lysR**	LysR family transcriptional regulator	WP_001050865.1	34 kDa	0.006	0.58	1.494849249
**cycA**	D-serine/D-alanine/glycine transporter	WP_000228322.1	51 kDa	0.014	0.58	1.494849249
**argT**	Lysine/arginine/ornithine ABC transporter substrate-binding protein ArgT	WP_000754418.1	28 kDa	0.018	0.57	1.484523571
**btuB**	TonB-dependent vitamin B12 receptor BtuB	WP_000591405.1	68 kDa	0.00017	0.55	1.464085696
**rssB**	Two-component system response regulator RssB	WP_000193429.1	37 kDa	0.004	0.55	1.464085696
**yhjY**	Autotransporter domain-containing protein	WP_000192421.1	70 kDa	0.008	0.55	1.464085696
**hybE**	Hydrogenase-2 assembly chaperone	WP_000004950.1	18 kDa	0.003	0.54	1.453972517
**yqjK**	Hypothetical protein	WP_000095496.1	12 kDa	0.006	0.54	1.453972517
**potF**	Spermidine/putrescine ABC transporter substrate-binding protein PotF	WP_000125769.1	41 kDa	0.012	0.54	1.453972517
**tesA**	Multifunctional acyl-CoA thioesterase I/protease I/lysophospholipase L1	WP_001010573.1	23 kDa	0.00033	0.53	1.45
**fsa**	Fructose-6-phosphate aldolase	WP_135424667.1	24 kDa	0.00045	0.53	1.443929196
**qseG**	Two-component system QseEF-associated lipoprotein QseG	WP_001054239.1	28 kDa	0.006	0.53	1.443929196
**pgtE**	Omptin family outer membrane protease PgtE	WP_000716010.1	35 kDa	0.006	0.53	1.443929196
**arnC**	Undecaprenyl-phosphate 4-deoxy-4-formamido-L-arabinose transferase	WP_000458893.1	37 kDa	0.001	-0.84	0.558643569
**emtA**	Membrane-bound lytic murein transglycosylase EmtA	WP_000776974.1	22 kDa	0.006	-0.85	0.554784736
**sptP**	SPI-1 type III secretion system effector GTPase-activating protein SptP	WP_023227558.1	60 kDa	0.006	-0.86	0.550952558
**spaK**	SPI-1 type III secretion system chaperone SpaK	WP_001164066.1	15 kDa	0.006	-0.86	0.550952558
**flgA**	Flagellar basal body P-ring formation protein FlgA	WP_001194076.1	24 kDa	0.00011	-0.87	0.547146851
**lrhA**	Transcriptional regulator LrhA	lWP_000606287.1	35 kDa	0.0046	-0.87	0.547146851
**argF**	Ornithine carbamoyltransferase	WP_000103033.1	37 kDa	0.00046	-0.91	0.532185091
**pspE**	Thiosulfate sulfurtransferase PspE	WP_000913428.1	12 kDa	0.002	-0.92	0.52850902
**ampH**	D-alanyl-D-alanine-carboxypeptidase/endopeptidase AmpH	WP_000830784.1	42 kDa	0.006	-0.98	0.50697974
**WP_000700634.1**	Hypothetical protein	WP_000700634.1	8 kDa	0.015	-0.99	0.503477775

Gene / locus name	Protein Function	Accession Number	Molecular Weight	Permutation Test (p-value) Benjamini-Hochberg (p < 0.02596)	Log2 Fold Change	Fold Change
**cdaR**	CdaR family transcriptional regulator	WP_000929420.1	44 kDa	0.009	-1	0.5
**WP_065618791.1**	Phosphatase PAP2 family protein	WP_065618791.1	27 kDa	0.006	-1.01	0.496546248
**pduB**	Propanediol utilization microcompartment protein PduB	WP_001734087.1	21 kDa	0.001	-1.02	0.493116352
**fimZ**	Fimbria biosynthesis transcriptional regulator FimZ	WP_079901676.1	24 kDa	0.006	-1.05	0.482968164
**WP_001683480.1**	PTS sugar transporter subunit IIA	WP_001683480.1	17 kDa	0.006	-1.09	0.469761375
**fljA**	Phase 1 flagellin gene repressor FljA	WP_000389002.1	20 kDa	0.01	-1.1	0.466516496
**fdxH**	Formate dehydrogenase subunit beta	WP_000061599.1	32 kDa	0.001	-1.13	0.456915725
**WP_000750393**	YgdI/YgdR family lipoprotein	WP_000750393.1	8 kDa	0.006	-1.13	0.456915725
**yjgB**	NAD(P)-dependent alcohol dehydrogenase	WP_001727706.1	36 kDa	0.006	-1.15	0.450625231
**tusD**	Sulfurtransferase complex subunit TusD	WP_001268010.1	14 kDa	0.008	-1.15	0.450625231
**allD**	Ureidoglycolate dehydrogenase	WP_000703934.1	38 kDa	0.006	-1.21	0.432268616
**cutC**	Copper homeostasis protein CutC	WP_001185769.1	27 kDa	0.001	-1.22	0.429282718
**yciE**	DUF892 family protein	WP_140076443.1	19 kDa	0.002	-1.23	0.426317446
**prgK**	Type III secretion system inner membrane ring lipoprotein PrgK	WP_000621238.1	28 kDa	0.006	-1.23	0.426317446
**fimC**	Molecular chaperone FimC	WP_000935025.1	25 kDa	0.006	-1.24	0.423372656
**chaB**	Putative cation transport regulator ChaB	WP_001146392.1	9 kDa	0.006	-1.25	0.420448208
**mscL**	Mechanosensitive ion channel family protein	WP_061451022.1	43 kDa	0.006	-1.3	0.406126198
**fdnI**	Formate dehydrogenase-N subunit gamma	WP_079959237.1	26 kDa	0.018	-1.31	0.40332088
**ubiH**	2-octaprenyl-6-methoxyphenyl hydroxylase	WP_000111128.1	42 kDa	0.012	-1.37	0.386891248
**pagN**	Adhesin/invasin protein PagN	WP_000787603.1	26 kDa	0.006	-1.41	0.376311687
**asmA**	AsmA family protein	WP_023227411.1	75 kDa	0.006	-1.43	0.371130893
**WP_000854956.1**	GntR family transcriptional regulator	WP_000854956.1	28 kDa	0.006	-1.43	0.371130893
**srlE**	PTS glucitol/sorbitol transporter subunit IIB	WP_000199033.1	34 kDa	0.005	-1.51	0.351111219
**mngR**	GntR family transcriptional regulator	WP_000572571.1	27 kDa	0.001	-1.54	0.343885455
**pepE**	Dipeptidase PepE	WP_000421776.1	25 kDa	0.018	-1.55	0.341510064
**sipA**	SPI-1 type III secretion system effector SipA	WP_023227560.1	74 kDa	0.00029	-1.57	0.336808394
**WP_001240360**	Membrane protein	WP_001240360.1	39 kDa	0.00017	-1.6	0.329876978
**sopD2**	Type III secretion system effector SopD2	WP_001145570.1	38 kDa	0.006	-1.6	0.329876978
**roP**	Rop family plasmid primer RNA-binding protein	WP_000165985.1	7 kDa	0.00049	-1.76	0.295248165
**pduD**	Propanediol dehydratase medium subunit PduD	WP_000405048.1	24 kDa	0.006	-1.77	0.293208737
**hemX**	Uroporphyrinogen-III C-methyltransferase	WP_000138954.1	42 kDa	0.001	-1.83	0.281264621
**WP_000826195**	DUF3617 domain-containing protein	WP_000826195.1	18 kDa	0.00011	-1.85	0.277392368
**dnaE**	DNA polymerase III subunit alpha	WP_001294818.1	130 kDa	0.006	-1.87	0.273573425
**srlD**	Sorbitol-6-phosphate dehydrogenase	WP_076916619.1	28 kDa	0.001	-1.88	0.271683716
**kduD**	2-dehydro-3-deoxy-D-gluconate 5-dehydrogenase KduD	WP_000602485.1	27 kDa	0.006	-1.91	0.266092546
**WP_000438384.1**	Hypothetical protein	WP_000438384.1	13 kDa	0.006	-1.92	0.26425451
**pduE**	Propanediol dehydratase small subunit PduE	WP_001090597.1	19 kDa	0.006	-1.94	0.26061644
**WP_000336847.1**	DUF1496 domain-containing protein	WP_000336847.1	10 kDa	0.006	-1.97	0.255253031
**deoR**	Hypothetical protein	WP_001123709.1	35 kDa	0.0002	-1.99	0.251738888
**yodD**	YodD family peroxide/acid resistance protein	WP_000844798.1	9 kDa	0.006	-2.02	0.246558176
**WP_001120854**	Hypothetical protein	WP_001120854.1	24 kDa	0.006	-2.04	0.243163737
**moD**	Site-specific DNA-methyltransferase	WP_000910374.1	73 kDa	0.0056	-2.09	0.234880687
**lprI**	Hypothetical protein	WP_000825951.1	12 kDa	0.006	-2.1	0.233258248
**psiF**	Phosphate starvation-inducible protein PsiF	WP_134815255.1	13 kDa	0.00038	-2.16	0.223756268
**cspC**	Cold-shock protein	WP_000208507.1	8 kDa	0.006	-2.24	0.211686328
**WP_001269912.1**	Hypothetical protein	WP_001269912.1	38 kDa	0.006	-2.32	0.200267469
**WP_001095011**	Membrane protein	WP_001095011.1	58 kDa	0.006	-2.36	0.194791145
**WP_001727706.1**	Hypothetical protein	WP_000877758.1	24 kDa	0.006	-2.63	0.161544104

Gene / locus name	Protein Function	Accession Number	Molecular Weight	Permutation Test (p-value) Benjamini-Hochberg (p < 0.02596)	Log2 Fold Change	Fold Change
**WP_000106461**	Isochorismatase family protein	WP_000106461.1	24 kDa	0.00085	-2.64	0.160428237
**WP_000356546.1**	Hypothetical protein	WP_000356546.1	9 kDa	0.006	-2.79	0.144586023
**mbeD**	Mobilization protein MbeD	WP_029401705.1	9 kDa	0.006	-2.84	0.139660892
**suhB**	Inositol-1-monophosphatase	WP_000553467.1	29 kDa	0.006	-2.85	0.138696184
**purL**	Phosphoribosylformylglycinamidine synthase	WP_000970045.1	141 kDa	0.006	-2.98	0.126744935
**frlB**	SIS domain-containing protein	WP_023206877.1	37 kDa	0.006	-3.08	0.118257206
**WP_000748128**	EexN family lipoprotein	WP_000748128.1	11 kDa	0.006	-3.64	0.080214119
**WP_000010697**	Hypothetical protein	WP_000010697.1	22 kDa	0.00037	-3.67	0.078563336
**WP_001185769.1**	Transporter substrate-binding domain-containing protein	WP_023139385.1	27 kDa	0.006	-3.85	0.069348092
**ilvC**	Ketol-acid reductoisomerase	WP_000024932.1	54 kDa	0.00043	-3.93	0.065607293
**nfsB**	Oxygen-insensitive NAD(P)H nitroreductase	WP_000355870.1	24 kDa	0.006	-4.38	0.048027349
**ybgS**	Hypothetical protein	WP_000784381.1	13 kDa	0.006	-5.51	0.021944451
**resD**	Resolvase	WP_033550418.1	25 kDa	0.006	-9.6	0.001288582
